# Non-ablative Erbium (YAG) and Neodymium (YAG) Laser Treatment for Anal Incontinence and Vaginal Atrophy: A Case Study

**DOI:** 10.7759/cureus.55542

**Published:** 2024-03-05

**Authors:** Nobuo Okui, Tadashi Ikegami, C. Tamer Erel

**Affiliations:** 1 Urology, Yokosuka Urogynecology and Urology Clinic, Yokosuka, JPN; 2 Dentistry, Kanagawa Dental University, Yokosuka, JPN; 3 Diagnostic Imaging, Kanagawa Dental University, Yokosuka, JPN; 4 Obstetrics and Gynecology, Istanbul University, Cerrahpasa School of Medicine, Istanbul, TUR; 5 Gynecology, Yokosuka Urogynecology and Urology Clinic, Yokosuka, JPN

**Keywords:** vulvodynia swab test, vaginal health index score, st. mark's score, cleveland clinic florida fecal incontinence score, neodymium:yag, erbium:yag, vaginal atrophy, anal incontinence

## Abstract

In this case study, a 68-year-old woman with anal incontinence (AI) and vaginal atrophy (VVA), who did not respond to traditional treatments such as pelvic floor exercises or hormone therapy, underwent three sessions of laser treatment using RenovaLase (SP Dynamis; Fotona d.o.o., Ljubljana, Slovenia), which employs non-ablative Erbium:YAG and Neodymium:YAG lasers. Significant improvements were observed in the VVA symptoms, with AI being resolved. The Vaginal Health Index Score increased from 7 points at the initial assessment to 18 points at 12 months after treatment. Similarly, the Cleveland Clinic Florida Fecal Incontinence Score and St. Mark's Incontinence Score, initially at 4 points each, improved to 0 points, indicating resolution of incontinence symptoms. MRI results demonstrated vascular enhancement and growth in the anal sphincter, with the thickness of the internal anal sphincter slightly increasing from initial measurements to a maximum of 0.36 cm, and improvements in resting and squeeze pressures from 42 mmHg to 110 mmHg, respectively. These findings underscore the effectiveness of RenovaLase® laser treatment for VVA and AI symptoms, offering a novel option for pelvic floor health management in postmenopausal women, especially those resistant to the use of artificial devices for anal improvement. In the environment of hormonal decline after menopause, the atrophy of pelvic vessels leads to reduced blood flow. This situation, where a noticeable lack of blood flow occurs during pretreatment of the pelvic vessels, is addressed by laser treatment. This phenomenon has been named "re-canalization." This case suggests the potential of this therapy as an alternative for patients resistant to conventional methods involving the insertion of devices into the anus to improve fecal incontinence. Further research is needed to explore its potential benefits.

## Introduction

Anal incontinence (AI) is defined by the International Continence Society as the involuntary discharge of gas or feces in a liquid or solid form [1,2]. The occurrence of fecal incontinence varies widely, affecting 1.4 to 19.5% of adults, influenced by the specific group studied, how information is gathered, and the definitions used [3,4]. A correlation between AI and vaginal atrophy (VVA), which is the drying and thinning of vaginal walls post-menopause due to decreased estrogen levels, has been suggested [5]. Despite its high prevalence, many people do not seek treatment due to embarrassment, and less than half receive it [6]. Treatment choices range from surgery for serious cases to medication and physical therapy for less severe cases, although not all conditions can be fully cured [7].

Traditional treatments for AI and vaginal dryness often fall short due to each person's unique physical makeup and varying degrees of their symptoms. This emphasizes the importance of identifying alternative treatments, such as laser therapy, for symptoms that are resistant to standard methods. VVA, affecting 75.3 to 87.3% of all women, is common [8,9] and is related to Genitourinary Syndrome of Menopause (GSM) [10], which includes menopausal symptoms, such as vaginal dryness and urinary discomfort, encompassing a broader range of symptoms affecting the genital and urinary systems and driving many gynecological consultations. Additionally, these conditions are often associated with pelvic floor muscle-related diseases, which include conditions affecting pelvic support, such as incontinence and prolapse. AI, VVA, and GSM are all pelvic floor muscle-related diseases, with MRI studies reporting their pathophysiology [11].

We present the case of a 68-year-old female patient, who had moderate AI due to mucus leakage post-defecation at her first visit for VVA treatment, which did not improve with pharmacotherapy or pelvic floor rehabilitation [12]. However, vaginal and anal laser treatments (non-ablative Erbium:YAG and Neodymium:YAG lasers) improved both VVA and AI symptoms. Innovation in this combination has proven effective in enhancing vaginal blood flow and alleviating congestion-related symptoms, such as vulvodynia, post-breast cancer treatment [13]. Additionally, modifications to the laser irradiation site have been reported to reduce temporary fecal incontinence and vulvar pain, and have been shown to be effective in treating severe urinary incontinence among female athletes [14,15]. A notable case reported by Mitrofanoff and Sikorski demonstrates the potential of intra-anal non-ablative Er:YAG laser treatment to significantly improve symptoms of rectal prolapse and fecal incontinence, underscoring the versatility of laser treatments in managing complex pelvic floor conditions [14]. This case exemplifies the broader applicability of laser therapy beyond VVA, addressing AI and rectal prolapse, and thereby contributing to a holistic approach to pelvic floor health.

This case study highlights the potential of laser therapy, underscoring the need for promising alternative means to improve the quality of life (QoL) of patients suffering from AI and VVA, conditions that are often inadequately addressed by conventional treatments [8-15].

## Case presentation

A 68-year-old woman with a history of three vaginal deliveries sought medical attention for vaginal pain and prolapse sensation, along with mild fecal incontinence. These symptoms, which started at five years post-menopause (menopause at 55 years without subsequent hormone replacement therapy), had worsened over the past 12 months. She had a history of hypertension and was managed with medication, but no diabetes or notable family medical history. The patient desired improvement in the symptoms of fecal incontinence, which affected her daily life and social activities. Additionally, she hoped for relief from pain associated with VVA and improvement in her QoL.

Examination revealed atrophic changes in the vulva, thin and dry vaginal mucosa with mild inflammation, and decreased elasticity of the vaginal walls, which caused pain upon palpation. Mild fecal incontinence and mucus secretion from the anus were observed with atrophy of the anal sphincter. Atrophy of the anal sphincter, along with other atrophic changes, highlights the critical role of estrogen deficiency and aging in the health of postmenopausal women [16,17]. Incontinence involves the involuntary discharge of liquid, leading to embarrassment and lowered self-esteem. No external or internal hemorrhoids were observed. Abdominal ultrasonography revealed no abnormalities, and colonoscopy revealed no significant lesions. Blood tests showed no anemia, and other values were within normal ranges. The Vaginal Health Index Score (VHIS) was 7, and the vulvodynia swab test averaged 4.5 points across the four locations [13]. Microscopic examination of the vaginal discharge revealed cellular atrophy and decreased lactobacilli [13]. The Cleveland Clinic Florida Fecal Incontinence Score (CCFIS) [18] for liquid stool incontinence was 4 points more than once a day St. Mark's score for liquid stool incontinence was 4 points, and daily use of pads (for protection against soiling from incontinence) scored 2 points [19,20]. The Digital Rectal Examination Scoring System had a resting score of 3 (normal 3) and a squeeze score of 3 (normal 3) [18]. In anorectal manometry, the resting pressure is 42 mmHg (normal range 40-70 mmHg) and the squeeze pressure is 102 mmHg (normal range 100-180 mmHg) [18]. Based on these findings, the patient was diagnosed with atrophic vaginitis and mild-to-moderate fecal incontinence.

For VVA, local estrogen therapy (LET) and the use of vaginal moisturizers were advised, with lubricants recommended to ease dyspareunia. However, after three weeks, the improvement in symptoms was minimal, and LET was discontinued due to induced headaches. The intake of polycarbophil calcium and loperamide hydrochloride was not effective for AI [13]. Additionally, pelvic floor muscle training (PFMT) was instructed for AI, but no improvement in fecal incontinence was observed with PFMT [13]. Despite efforts to manage symptoms and improve QoL through pelvic floor strengthening, hormone therapy, diet, and lifestyle changes, no significant results were achieved at three months. For severe cases, surgical options are available, but there were no suitable treatments for moderate to mild severity, and the patient was reluctant to pursue sacral nerve modulation because of the insertion of an artificial device and potential exacerbation of pain [18].

The patient prioritized treating VVA first. Vaginal Erbium YAG (VEL) and Neodymium YAG (Nd:YAG) laser treatment improved vaginal pain and health index, with concurrent fecal incontinence improvement observed after the first session (L1). This treatment approach is supported by the literature demonstrating the efficacy of Er:YAG and Nd:YAG lasers in managing genitourinary syndrome and rectal prolapse symptoms, respectively [10,14]. This combination is known to promote collagen production and tissue regeneration. Additionally, anal Erbium:YAG laser (AEL) treatment was introduced (L2), showing significant effectiveness, and after the third session of VEL+AEL+Nd:YAG (L3), VVA greatly improved and fecal incontinence was completely resolved. After a year, the patient remained well but underwent a fourth session (L4) due to recurrence concerns, as per the patient’s request. MRI and ultrasound were conducted 18 months post-treatment (T18). Laser procedures were outpatient, with pre-treatment vaginal disinfection and drying. The RenovaLase® protocol utilized a 2,940 nm wavelength, with specific steps for VEL and Nd:YAG laser application, including parameters for vaginal and anal treatments. Informed consent was obtained from the patients undergoing laser treatment, and an agreement was signed.

**Figure 1:**
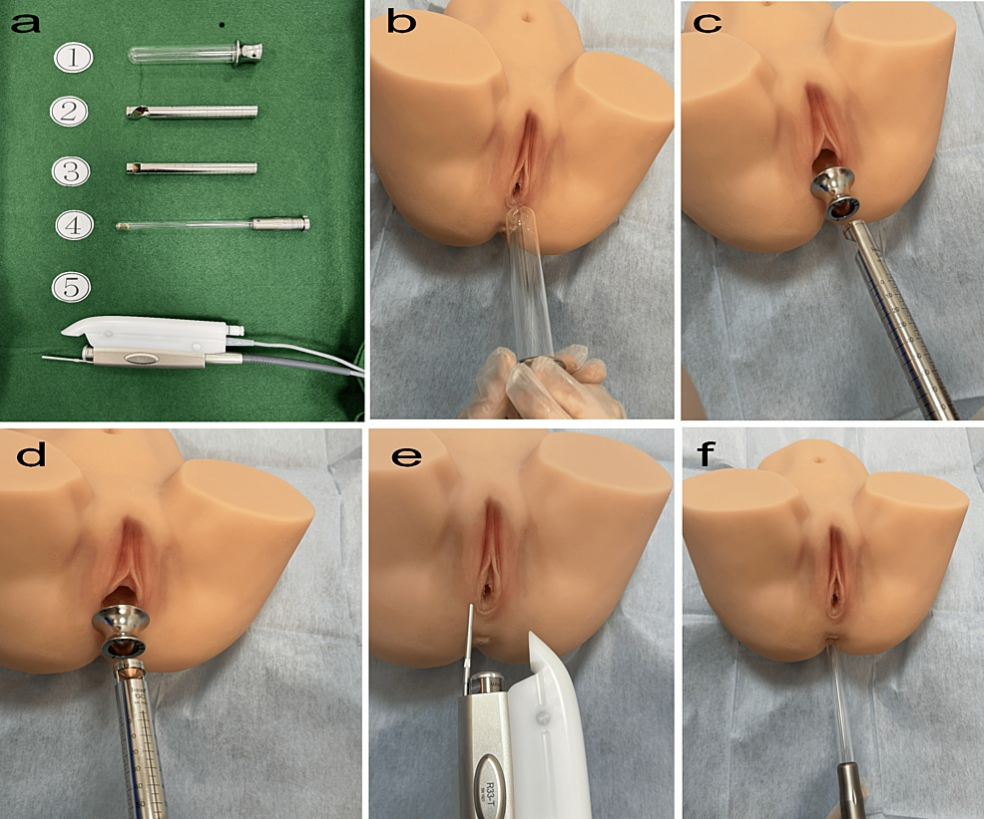
VEL+NdYAG+AEL treatment procedure a. SP Dynamis; Copyright © 2013. Provided courtesy of Fotona d.o.o. (Ljubljana, Slovenia). This image in Figure 1a is provided free of charge by Fotona d.o.o. :. 1, Special glass speculum for laser; 2, PS03-GA angular adapter laser probe for the anterior wall of the vagina; 3, R11-GC circular adapter laser probe for the entire circumference of the vagina; 4, SmoothTouch LA Adapter probe for the entire circumference of the anus; 5, R33 NdYAG laser probe for vulvar irradiation. b. VEL step (glass speculum insertion). c. VEL step (laser irradiation of posterior vaginal wall by PS03-GA). d. VEL step (whole vaginal laser irradiation by R11-GC1). e. Nd:YAG laser (Fotona SP Dynamis, PIANO mode, spot size 9 mm, R33 non-contact handpiece, PIANO pulse mode (five seconds), fluence 90 J/cm^2^). f. AEL step (circumferential intra-rectal ErYAG irradiation with LA adapter). AEL: Erbium:YAG laser; VEL: Vaginal Erbium YAG; NdYAG: Neodymium YAG

Figure 1 shows model images of the VEL, Nd:YAG, and AEL lasers. The laser treatment was performed on an outpatient basis. Before the procedure, the patient's vagina was cleaned with disinfectant and dried with a cotton swab. The RenovaLase® (SP Dynamis; Fotona d.o.o., Ljubljana, Slovenia) VEL protocol used a wavelength of 2,940 nm.

Step 1 involved VEL with an angular adapter and PS03 handpiece at 10.00 J/cm², 2.0 Hz, and 7 mm spot size, applied in six segments three times to the vaginal posterior wall.

Step 2 uses VEL with a circular adapter and R11 handpiece at 3.00 J/cm², 2.0 Hz, and 7 mm spot sizes, covering the entire vagina in six segments once.

The 1064 nm Nd:YAG laser treatment was administered in a continuous PIANO pulse mode for a duration of 30 minutes, utilizing a non-contact R33 handpiece set at an energy density of 90 J/cm², with a five-second pulse duration, and a 9 mm spot size, targeting the perineum and external genitalia.

For the anal step, AEL treatment measures the anal sphincter width via ultrasound before inserting the SmoothTouch LA Adapter attached to the R11 handpiece, applying 3.00 J/cm², 2.0 Hz, 7 mm spot size in six segments once over the entire anal area for two minutes.

**Figure 2:**
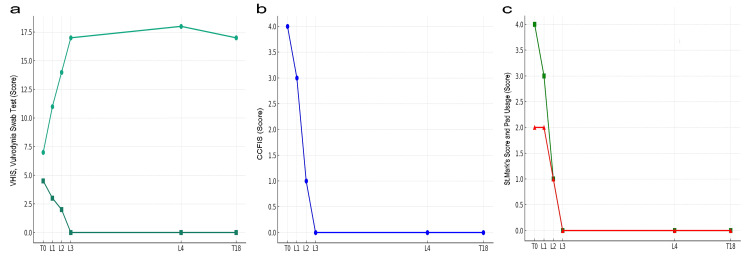
The changes before and after laser treatment a: VHIS (Vaginal Health Index Score, green closed circles) and vulvodynia swab test (green closed squares), b: CCFIS (Cleveland Clinic Florida Fecal Incontinence Score, blue closed circles), c: St. Mark’s score (green closed circles) and pad usage (red closed circles). Notations include T0 for initial consultation (pre-treatment), T18 (18 months after the third laser treatment, L3), and L1, L2, L3, and L4 indicating the first, second, third, and fourth laser treatments, respectively.

Figure 2 shows the progression and results of the various questionnaires. At the initial consultation (T0), VHIS was 7 points, vulvodynia swab test 4.5 points, CCFIS for liquid stool incontinence was 4 points, St. Mark's score for liquid stool incontinence 4 points, and pad usage 2 points. MRI and ultrasonography were performed at T0. One month post-L1, improvements were noted: VHIS 11, vulvodynia swab test 3, CCFIS 3, and St. Mark's score 3, and pad usage 2 points. After implementing L2 upon the patient's suggestion for direct Er:YAG laser application to the anus and reassessing one month later, further improvements were observed: VHIS 14, vulvodynia swab test 2, CCFIS 1, St. Mark's score 1, and pad usage 1 point. Following the same protocol as L2, L3 led to significant improvements one month later: VHIS 17, vulvodynia swab test 0, CCFIS 0, St. Mark's score 0, and pad usage 0 points. One year after L3, the scores were maintained (VHIS 18, vulvodynia swab test 0, CCFIS 0, St. Mark's score 0, pad usage 0), but L4 was conducted at the patient's request. Six months after post-L4, the scores remained stable, and MRI and ultrasound were performed (T18). In the T18 anorectal manometry, the resting pressure was measured at 42 mmHg, and the squeeze pressure was measured at 110 mmHg, indicating a slight increase/no change in the measurements. L1 to L2 and L2 to L3 intervals were one month each, while the L3 to L4 interval was 12 months.

Following the completion of the initial three-set laser treatment program, with the last being L3, symptoms of VVA and fecal incontinence disappeared, and the patient experienced a sustained good QoL for one year. Six months after the additional laser treatment at L4, the patient was in a good state. L4 was performed because of the patient's strong concerns about recurrence, despite not being explicitly recommended by the doctor. The patient reported high satisfaction with the treatment, no side effects, and improvements in daily activities such as reduced fear of going out and using public transport.

Additionally, while the initial outcomes were promising, assessing the durability of the results through long-term follow-up is crucial. Monitoring the patient's condition over an extended period of time will provide valuable insights into the sustained efficacy of laser treatment and potential symptom recurrence beyond the one-year observation period. Therefore, collecting long-term data is essential for the comprehensive evaluation of treatment efficacy and safety over time.

**Figure 3:**
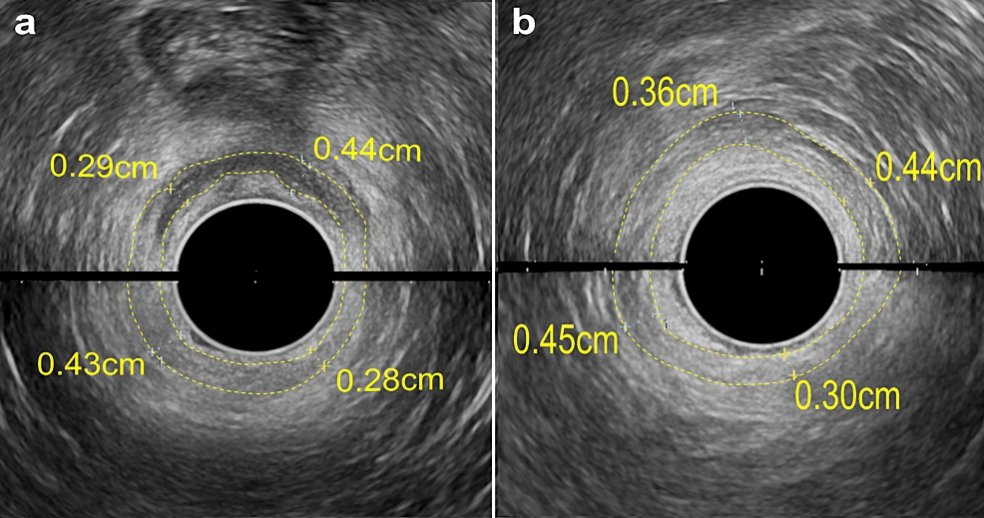
Anal ultrasound images Using a 180-degree probe, each image showed the anterior (top) and posterior (bottom) aspects. Image a shows pre-treatment (T0), and image b shows post-treatment (T18). The yellow dotted lines indicate the internal anal sphincter as detected by ultrasound. Measurements of sphincter thickness at four equidistant points, using the perineum as the 12 o'clock reference and moving clockwise, are provided in centimeters (cm).

Figure 3 presents an anal ultrasound using an Afietta65 (Fujifilm Co., Tokyo, Japan) with a 180-degree probe, taken in two parts (upper and lower). The thickness of the internal anal sphincter was uniformly measured at four points around the perineum, designated as 12 o'clock. At T0, ultrasound measurements were 0.44 cm, 0.28 cm, 0.43 cm, and 0.29 cm. By T18, these measurements slightly increased to 0.44 cm, 0.30 cm, 0.45 cm, and 0.36 cm, indicating a minor growth in the sphincter's thickness.

**Figure 4:**
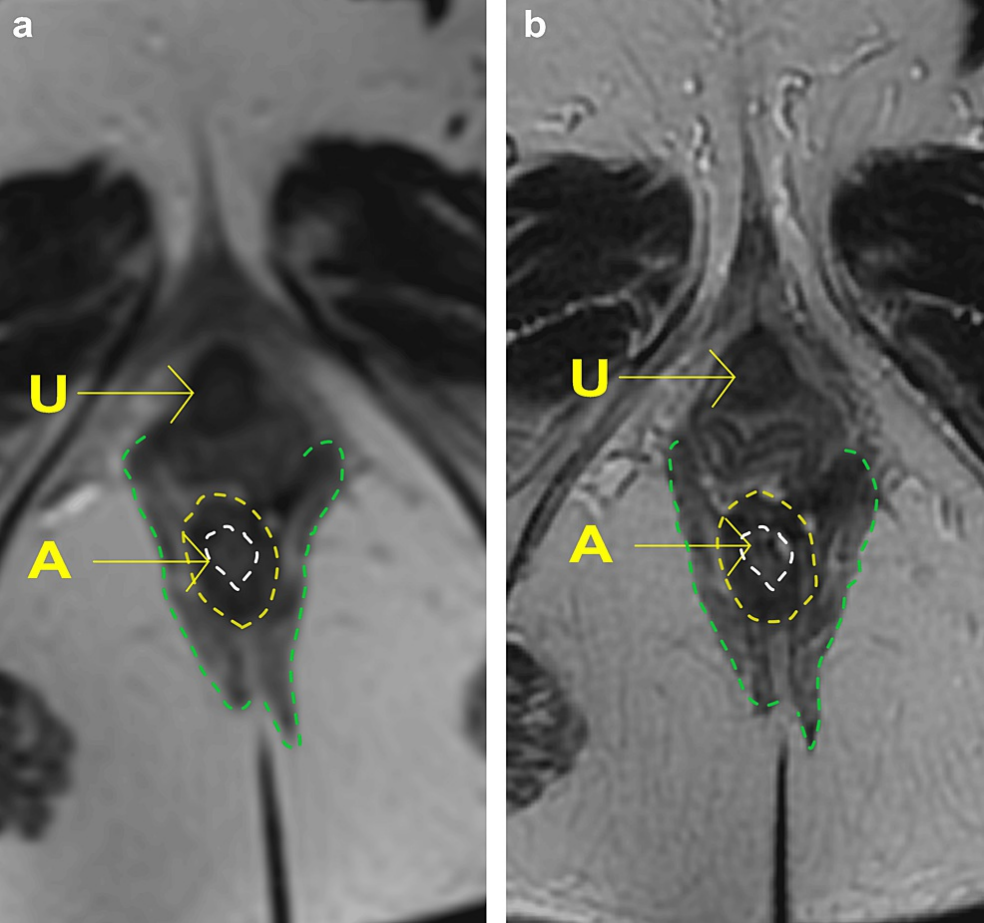
The anal sphincter muscles The T2-weighted sagittal view MRI using a 1.5T MRI (Signa Creator, GE Healthcare, Chicago, USA). The images, with specified MRI parameters, show a comparison before (a: T0) and after (b: T18) treatment, with the urethra (U) and the anus (A) indicated by arrows. The yellow dotted lines identify the internal anal sphincter as seen on MRI, the green dotted lines represent the external anal sphincter, and the white lines outline the contour of the anus.

The patient underwent MRI analysis of the pelvic organs before and after treatment using a 1.5T MRI (Signa Creator, GE Healthcare, Chicago, USA). Figure 4 shows T2-weighted sagittal view MRI images (TR4500 ms, flip angle 140 degrees, TE 100 ms, slice thickness 3 mm, interval 3.5 mm, FOV 32x36 cm, Matrix 320x320). It compares the external and internal anal sphincter muscles at T0 (Figure 4a) and T18 (Figure 4b) and shows similar sizes at T0 and T18.

**Figure 5:**
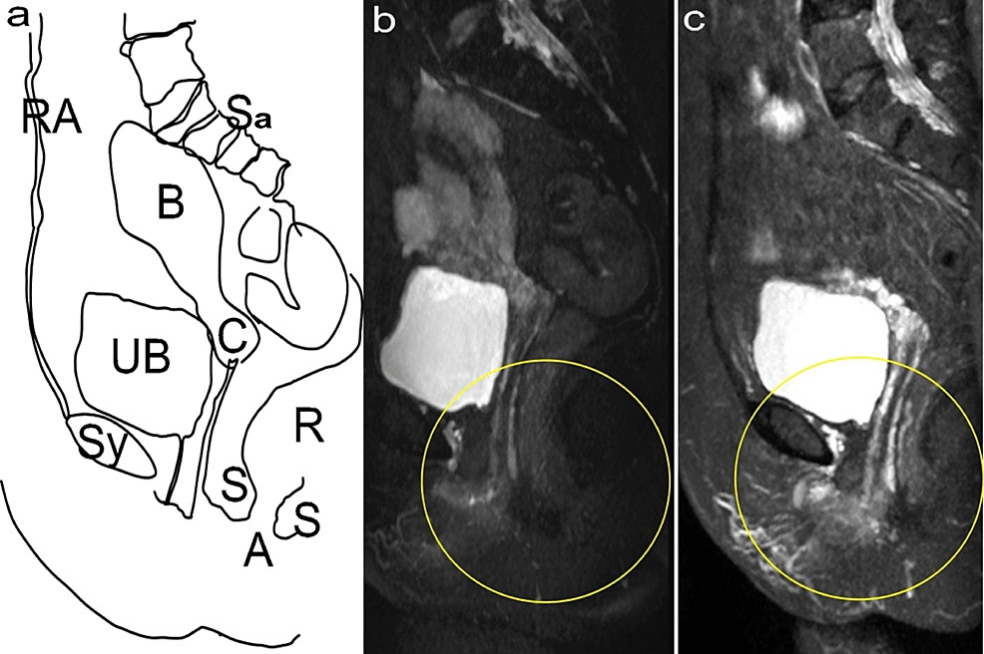
Changes in sagittal images before and after treatment a: Illustration of organ names. b: before treatment (T0); c: after treatment (T18). The yellow circles indicate re-established blood flow within the blood vessels due to laser therapy. Credit: Illustration by the author (Nobuo Okui). RA: Rectus abdominis; Sy: Symphysis pubis; UB: Urinary bladder; B: Uterine corpus; C: Cervix of the uterus; A: Anus; S: Anal sphincter; R: Rectum; Sa: Sacrum

Figure 5 displays MRI images reconstructed to sagittal and axial views with an 8-mm thickness after maximum intensity projection (MIP) treatment. These images were derived from the 3D data acquisition of T2-weighted coronal images with fat suppression, showcasing detailed imaging parameters. The areas encircled in yellow highlight regions where, at T0, no blood flow was detected in the vessels, indicating an unhealthy state. At T18, these same regions showed a healthy state with maintained blood flow, illustrating significant improvement and restoration of vascular health.

**Figure 6:**
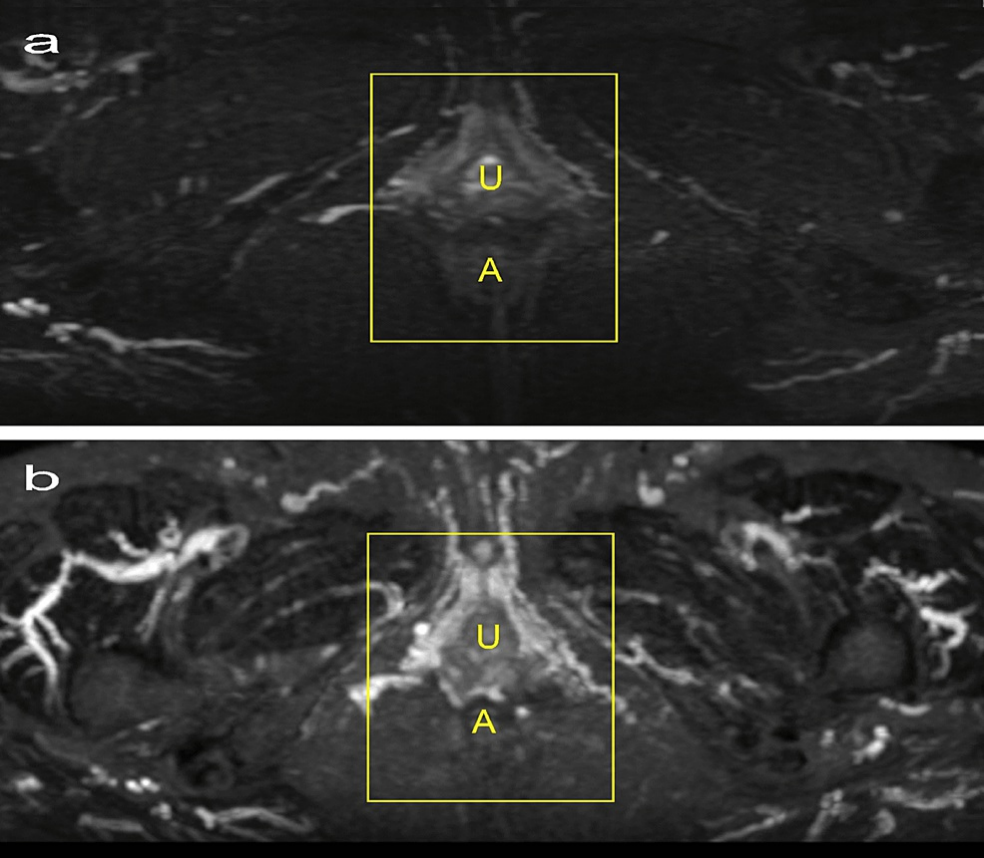
Restoration of blood flow in the urethra, vagina, and anus by ROI interest 1.5T MRI (Signa Creator, GE Healthcare, Chicago, USA). MRI was reconstructed into sagittal and axial 8-mm-thickness images after MIP treatment using 3D data acquisition of T2-weighted coronal images with fat suppression (TR 3500 ms; TE 100 ms; inversion time 150ms; flip angle 90°; FOV 42 × 48 cm; matrix 384 × 256). Each image depicts the upper (cranial) and lower (caudal) aspect. a: Before treatment (T0), b: After treatment (T18). U: Urethra, A: Anus. The yellow lines represent the ROI. ROI: Region of interest; MIP: Maximum intensity projection; FOV: Field of view; TR: Repetition time; TE: Echo time

Figure 6 shows a comparison of the areas with improved blood flow, as shown in Figure 5. Using the same MRI conditions, the evaluation of vascular density within a rectangular area, set in proportion to the distance between the urethra and anus in axial slices, was based on the coefficient of variance. This metric, calculated by dividing the standard deviation by the mean signal intensity in the specified region of interest (ROI), allows for quantitative assessment of vascular density uniformity and variability within the ROI. The signal-to-noise ratio (S/N ratio) at T18 was 2.10, compared with 1.56 at T0, enabling the evaluation of signal quality and measurement precision changes over the observation period.

## Discussion

This case report focuses on a 68-year-old woman who underwent laser treatment for VVA and AI and highlights the crucial connection between urogenital and colorectal health. Despite the limited existing literature that simultaneously addresses VVA and AI, this case illustrates how postmenopausal estrogen deficiency can affect these conditions. Previous research has established the consequences of estrogen deficiency following menopause, emphasizing the significant role hormonal changes play in health conditions related to both the urogenital and colorectal systems [5]. This underscores the necessity for a comprehensive approach to pelvic region care in postmenopausal women's health, going beyond the traditional methods.

Further research is necessary to deepen our understanding of the relationship between AI and VVA as well as how these conditions affect each other. It is crucial to investigate the reasons for the effectiveness of the laser treatment under both conditions. This process involves examining changes in three key areas: (1) vascular, (2) muscular, and (3) mucosal. (4) In addition, a comprehensive summary of previous research on laser treatment is required. These steps are essential for gaining a comprehensive understanding of the effectiveness of treatment and the interconnectedness of these conditions.

First, vascular changes will be examined using MRI, as advocated in previous studies [11]. This MRI clearly showed that vessels, which had significantly reduced blood flow due to menopause, resumed stable blood flow over a prolonged period. Concurrently, improvements in the VHIS and vulvodynia swab test, indicators of VVA symptoms, suggest that stable blood flow within these vessels has led to enhanced metabolic activity and tissue regeneration. In clinical practice, it is common to encounter intensified symptoms post-menopause, as in this patient, at T0. Previous research indicates that sex hormones, especially estrogen and testosterone, affect pelvic blood vessels post-menopause, potentially leading to reduced blood flow velocity and increased resistance indices [21]. This lack of blood flow during pelvic vessel pretreatment was evident. Laser treatment, known for its distinctive action of vasodilation and reperfusion, has been described as "re-canalization" [22], a term also applicable to the findings of this study.

Second, muscular changes resulting from the laser treatment did not show any distinct alterations. Anal ultrasonography revealed only minor changes in size, while MRI showed little to no significant changes before and after treatment. There has been no prior investigation of MRI analysis in the treatment of AI using energy devices, leaving it uncertain whether the anal sphincter muscles could regenerate in this case. In instances of urinary incontinence, investigations utilizing MRI have shown that laser treatment does not lead to regeneration or thickening of the urethral sphincter muscle [15]. Instead, it improved the coordinated movements of the entire pelvic floor muscles when intentionally tightened, subsequently leading to an improvement in urinary incontinence [15]. A similar rationale could be applied in the case of fecal incontinence. That is, laser treatment may not directly stimulate the regeneration of the anal sphincter muscles but could potentially improve the condition of fecal incontinence by enhancing the coordination and function of the pelvic floor muscles.

Third, mucosal changes were indicated by VHIS and vulvodynia swab tests. In this case, as shown in previous research, the combination of a VEL and Nd:YAG laser for VVA demonstrated effectiveness [10]. According to numerous studies, VEL or VEL + Nd:YAG laser treatment induces reconstitution of collagen and neovascularization in the mucosa, transforming the thin mucosa of patients with VVA into the thickness of healthy women's mucosa [10]. This is particularly beneficial for breast cancer survivors who are unable to undergo LET [13]. In this case, the patient had severe side effects, such as headaches from the LET, making laser treatment a valuable alternative. Previous research has shown that fecal incontinence can be improved by applying a device (non-excisional circumferential radiofrequency) to the anus using non-ablative energy [2]. What our laser treatment shares in common is its non-ablative nature. Non-ablation, which does not damage the tissue surface, is believed to be effective for tissue reconstitution.

Finally, a summary of previous studies on laser treatment and AI improvement was presented in this case. Because anal incontinence is the leakage of gas or feces due to the inability to meet the increase in intrarectal pressure due to insufficiency of the anal sphincter [23], it is necessary to consider how the VEL, Nd:YAG laser, and AEL laser may strengthen the anal sphincter. VEL, especially the Er:YAG laser applied to the posterior vaginal wall with either an angular or circular head, can regenerate the connective tissue in the epithelium and provide elasticity to the ampulla recti [24].

This flexibility may reduce the pressure in the ampulla recti. In contrast, Nd:YAG laser applied to the perineum can increase the strength of the anal sphincter by increasing the tone of the perineal muscles [13]. Moreover, by acting on deeper layers, it may reduce the pressure in the ampulla recti by showing its regenerative effect on the tone of the puborectal muscle of the levator just above and deeper than the anal sphincter. AEL application may have had a regenerative effect on both the anal sphincter and the puborectal muscle. This thermochemical effect may occur via neoangiogenesis. In particular, the anatomical neighborhood of the rectum and vagina may have enabled the Er:YAG laser treatment to have a positive effect on anal sphincter insufficiency [14]. The anterior vaginal wall is thicker than the posterior vaginal wall [25].

This thickness was due to the collagen content of the anterior vaginal wall. Similarly, the collagen content in the posterior vaginal wall predicts thickness. The non-ablative Er:YAG laser primarily targeted the collagen content of the epithelium. In contrast, the proximal portion of the posterior vagina was claimed to have richer connective tissue than the distal portion [26].

Moreover, this variation in the connective tissue of the posterior vaginal wall may predict the endorectal pressure. Therefore, the fact that the Er:YAG laser affects superficial tissues in the posterior vaginal wall and the Nd:YAG laser affects deeper tissues in the perineum provides us with an explanatory mechanism that can be used in the treatment of anal incontinence [13]. However, the presence of estrogen receptors in the anal sphincter, anal mucosa, and rectal mucosa and the changes in this region after menopause and menopausal hormone therapy for rectal incontinence is controversial [17,27]. However, among the regenerative effects of lasers, the local production of estrogen receptors is among the topics that have drawn recent attention. Proving this situation will be a mechanism that will explain the treatment of anal incontinence as a component of pelvic floor dysfunctions, as in urinary incontinence [28].

## Conclusions

This case study suggests that laser treatment may be an effective alternative for patients who do not respond to traditional treatments and benefits women resistant to the idea of implanting foreign objects, such as SNM devices. Additionally, women with VVA or GSM may have underlying AI. Owing to embarrassment, patients may not be able to share information about AI. It is essential to comprehensively consider patients and explore how such noninvasive approaches can aid in the development of future treatment methods. Regarding AI, as some previous studies have suggested, options include lifestyle improvements, dietary changes, and medication. Laser treatment, with its distinctive action of vasodilation and reperfusion, can be used as the next step in treatment. This approach, described as "re-canalization," addresses the lack of blood flow during pelvic vessel pretreatment, illustrating how laser treatment's unique mechanisms of action contribute to its effectiveness in treating conditions like AI by enhancing tissue perfusion and promoting healing.
